# Confidential enquiries into maternal deaths in Ondo State, Nigeria – a comparative analysis

**DOI:** 10.1186/s12884-019-2659-y

**Published:** 2019-12-21

**Authors:** Lawal Oyeneyin, Thomas van den Akker, Oladipo Durojaiye, Ola Obaado, Francis Akanbiemu, Yetunde Olagbuji, Isaac Aladeniyi, Majeed Oyeneyin, Olajumoke Aladenola

**Affiliations:** 1 Mother & Child Hospital Ondo, Ondo, Ondo State Nigeria; 2 Department of Obstetrics and Gynaecology, Leiden University Medical Centre, Leiden, and Athena Institute, Vrije Universiteit, Amsterdam, Netherlands; 3 Department of Planning, Research & Statistics, Ministry of Health, Akure, Ondo State Nigeria; 4 Department of Planning, Research & Statistics, Hospitals Management Board, Akure, |Ondo State Nigeria; 5 Department of Planning, Research & Statistics, Primary Healthcare Development Board, Akure, Nigeria

**Keywords:** Confidential enquiries, Maternal deaths, Maternal mortality ratio

## Abstract

**Background:**

Paucity of data on state-wide maternal mortality in Nigeria hampers planning, monitoring and evaluation of the impact of interventions. The Confidential Enquiry into Maternal Deaths in Ondo State was initiated to overcome this problem. This study aimed to compare trends of maternal mortality ratios, causes of deaths, geographical distribution and other associated factors in 12-monthly reports of the Confidential Enquiry into Maternal Deaths in Ondo State.

**Methods:**

Notification forms were distributed throughout the State to focal persons and medical records officers at community and facility levels, respectively. Maternal deaths, as defined in the International Classification of Diseases 10th version, were recorded prospectively over 3 years from 1st June 2012 to 30th May, 2015. Forms were submitted, collated and data analysed by a multidisciplinary review committee.

**Results:**

Reported numbers of maternal deaths (and maternal mortality ratios) were 114 (253 per 100,000 births), 89 (192) and 81 (170), respectively per year, indicating a 33% reduction in maternal mortality ratio over the course of the study period. Assuming that the confidential enquiry process was the only intervention at the time aimed at reducing maternal mortality, simple linear regression with a correlation coefficient of 0.9314, showed a relationship though the difference in the values were not statistically significant (95% CI = − 184.55 to 101.55, *p* = 0.169). Postpartum haemorrhage and eclampsia were the leading causes of deaths.

**Conclusion:**

There was a trend of reduction in maternal mortality ratio during the period of study with postpartum haemorrhage as the major cause of death. The positive association between the confidential enquiry reports and maternal mortality ratios make us recommend that our model be adopted in other states and at the federal level.

## Introduction

Maternal death is defined as “the death of a woman while pregnant or within 42 days of termination of pregnancy, irrespective of the duration and site of the pregnancy, from any cause related to or aggravated by the pregnancy or its management, but not from accidental or incidental causes” [1]. Globally, an estimated 303,000 maternal deaths occurred in 2015 with low- and middle-income countries accounting for 99%. At the country level, Nigeria and India accounted for a third of these maternal deaths with 58,000 (19%) and 45,000 (14%), respectively [2]. The maternal mortality ratios (MMR) in Nigeria were estimated to be 545 and 576 (per 100,000 live births) in 2008 and 2013, respectively [3]. These figures have a wide range depending on the region of the country. The states in the northern regions like Zamfara, Bauchi, Kano, etc. have worse health indices due to over population and less economic development when compared to those in the south like Lagos, Ondo, Anambra, etc. Identifying accurate information on specific circumstances surrounding the death of women in countries with inadequate vital registration statistics like Nigeria would enable efforts to reduce maternal mortality [4].

Confidential enquiries into maternal deaths are one type of maternal death surveillance and response, which intend to go beyond counting numbers of deaths for statistical purposes, and try to provide insight into possible factors that may be addressed to reduce future deaths. Confidential enquiry allows for analysis of what could be done in practical terms at facility, community and state levels [4]. The earliest reference to a maternal death surveillance system was in Sweden in 1660 which eventually led to presentation of the first national statistics on maternal mortality in 1751 [5]. The first modern-day formal reporting of a confidential enquiry covered England and Wales for the years 1952–1954, although the system on which it was based had been established as early as 1928 [6].

In October 2009, multi-pronged health interventions and strategies under the umbrella of the “Abiye” (“safe motherhood”) programme were promoted and implemented in Ondo State. They were to find solutions to the four phases of delay predisposing to maternal and child deaths i.e. delays in seeking, reaching, accessing and referring care [7]. The absence of accurate state-wide maternal health indicators, however, made it difficult to appropriately plan, monitor and evaluate the impact of these interventions and strategies. Several pragmatic steps were then taken to legislate the Confidential Enquiry into Maternal Deaths in Ondo State (CEMDOS) on 24th of May, 2010 [7]. Data generated were presented to stakeholders and policy-makers periodically for deliberation. This study aimed to compare 12-monthly trends of maternal mortality ratios (MMRs), deaths and their geographical distributions, reported to the CEMDOS.

## Methods

### Study setting

Ondo State was founded in 1976 in south-west Nigeria. It has a land area of about 15,000 km^2^ with one of longest coast lines in the country. It is composed of 18 local government areas (LGAs) distributed across three senatorial districts and its capital is Akure. Though an oil producing State, it is a largely rural and agrarian society noted for cultivation of cocoa among other cash crops, as well as mineral deposits. The most recent National Census of 2006 recorded a population figure of 3,450,877 [8].

At the time of this study, Ondo State had 14 secondary-level general hospitals as well as seven tertiary referral centres, namely; four state-run specialist hospitals, two purpose-built maternities and a facility in Owo LGA run by the federal government. Two maternities and two of the state-run specialist hospitals are located in the highly populated Akure South and Ondo West LGAs. The remaining two state-run hospitals are in the less densely populated Akoko Northeast and Okitipupa LGAs. The general hospitals are evenly distributed among the remaining 14 LGAs. All hospitals offer maternity care and serve as referral facilities for the health centres in their respective catchment areas.

### Study design

This study was a comparative analysis of 12-monthly CEMDOS data over 3 years from 1st June 2012 to 31st May 2015. Data collection took place at both facility and community levels.

Collection of data involved distribution of customised maternal death notification forms to two medical records officers in each of the 21 designated hospitals mentioned above. Prior to distribution, medical records officers and medical directors of the hospitals were trained on facility-based maternal death reviews. Maternal death notification forms were completed in triplicates: one copy each for the CEMDOS secretariat, office of the chief medical-record-officer and the facility.

At community level, customised notification forms were distributed to focal persons who were, prior to the study, disease surveillance and notification officers with a minimum of completed secondary education statutorily responsible for reporting community-based diseases with public health impact e.g. poliomyelitis, cholera, etc. Two of the most experienced notification officers were selected for each LGA based on number of years spent as residents within their respective communities. Their activities were supervised by monitoring and evaluation staff whose offices were located in the headquarters of each LGA. Prior to data collection, both cadres underwent intensive training on conducting confidential verbal autopsies and filling of notification forms also in triplicates: one copy each for the CEMDOS secretariat (state level), monitoring and evaluation office (local government level) and custody of notification officers (community level). Throughout the process, care was taken to secure the completed forms containing sensitive personal data in files kept in locked drawers in the individual offices.

This bi-modal approach was used to ensure that in addition to hospital-based compilation, deaths occurring outside facilities were captured in order to increase the veracity of state-wide maternal death data. Concurrently, advocacy and sensitisation workshops were held in each LGA for key stakeholders including private and public sector healthcare providers, traditional as well as religious leaders. Jingles in local dialects creating awareness on the importance of accurate maternal death reporting were also produced on radio and television for mass media sensitisation. These activities ensured that the cooperation of members of the public in divulging relevant information was maximised.

Data on the maternal deaths included socio-demographic characteristics, place, date and time of death; suspected causes (in the absence of confirmation post-mortems) as well as any special circumstances surrounding the death, as determined by the trained data collectors. All were recorded prospectively over the period of study. Within a week of completing each form in triplicate, copies were submitted by the monitoring and evaluation as well as chief medical records officers to the CEMDOS secretariat domiciled in the state ministry of health. Fortnightly, the CEMDOS secretary collated the forms ensuring that double reporting was excluded for final vetting. The members of the CEMDOS committee comprising multi-disciplinary maternal health experts and advocates conducted the critical examination process on a monthly basis, according to the principles of a confidential enquiry as practised in some countries [9]. All authors except one external expert were physician members of this committee and employees of the Ondo State government at the time of study.

Further information required on any reported case was pursued individually by reaching out to the focal persons or contacts of the deceased. At this stage, causes of deaths were also coded using the WHO International Classification of Diseases to ensure standardisation. To allow for inputs and feedback on counter strategies, preliminary 6-monthly reports were presented to the head of the ministry in the presence of stakeholders from the communities and hospitals.

### Data analysis

In the absence of accurate vital registration statistics, the figures for the denominator in calculating annual maternal mortality ratio were extrapolated from the crude birth rates which in turn were derived from the estimated number of live births and the mid-year population of Ondo State taking into cognisance the annual growth rate of 3% [8]. The data were entered into SPSS software; a correlation analysis was used to represent the relationship between the CEMDOS intervention and the trend of MMR, as determined by simple linear regression. Fisher’s exact test was used to ascertain significant difference (*p* < 0.05 and 95% confidence level) for other relevant variables.

It is acknowledged that the preferred denominator in general usage for calculating MMR is number of “live births” rather than “total births” (which should include stillbirths). In Nigeria, stillbirths tend to go unrecorded when compared to live births. For this study however, the denominator was total births having established that the omission or commission of stillbirths in MMR calculations was previously judged as being relatively insignificant [10].

## Results

The figures of consecutive annual births were 45,000, 46,350 and 47,700 in 2012, 2013 and 2014, respectively. The numbers of reported maternal deaths and MMRs (per 100,000 births) during the periods of study are shown in Table 1. This amounts to a 33%-reduction in MMR in the period of study. Assuming that the confidential enquiry process was the only intervention at the time aimed at reducing maternal mortality, simple linear regression with a correlation coefficient of 0.9314, showed a positive relationship. The difference in the values, however, were not statistically significant (95% CI: − 184.55 to 101.55, *p* = 0.169).

**Table 1:** Breakdown of estimated births, maternal deaths and maternal mortality ratios

CEMDOS Period of Report (12 monthly)	Total Estimated Births	Number of Reported Maternal Deaths	Maternal mortality r atio (per 100,000 births)
1st June, 2012 – 31st May, 2013	45,000	114	253
1st June, 2013 – 31st May, 2014	46,350^a^	89	192
1st June, 2014 – 31st May, 2015	47,700^a^	81	170

^a^taking into cognizance annual population growth rate of 3%

**Fig. 1:**
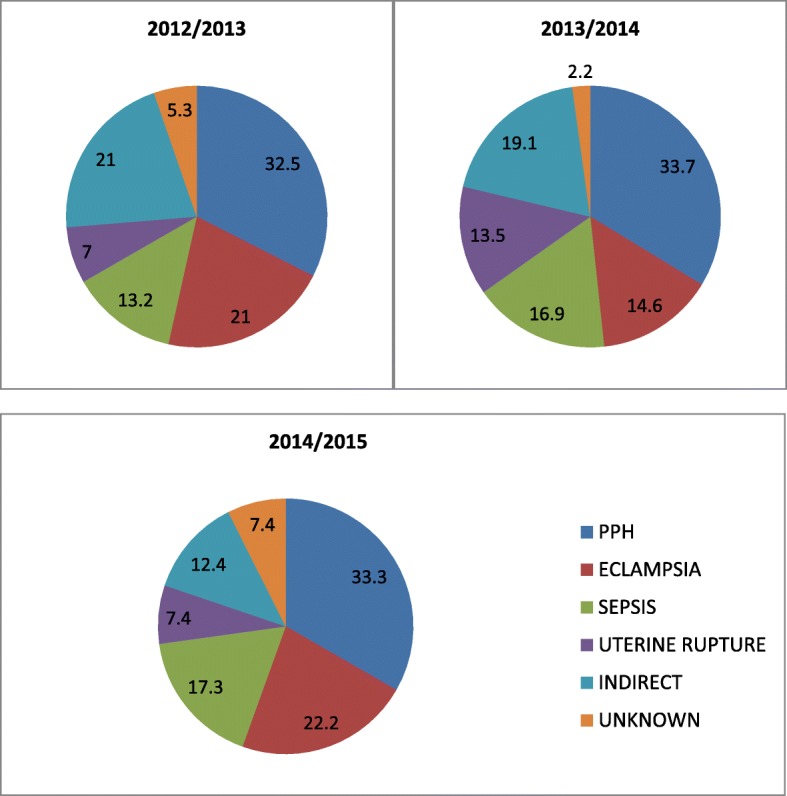
Distribution of Causes of Maternal Deaths

Postpartum haemorrhage (PPH), eclampsia, septicaemia and uterine rupture were consistently reported as the leading causes of maternal deaths throughout the study period. The indirect causes were individual cases of medical conditions in pregnancy like suspected pulmonary embolism, severe hepatitis, diabetic keto-acidosis, pneumonia, congestive heart failure, sickle cell anaemia, severe acute asthma and anaesthetic death. Figure 1 illustrates these major causes.

About 90% of the women died in public or private hospitals, irrespective of the duration of time spent on admission. Further enquiry by the data officers, however, showed that over 80% of those facility deaths were women who had not booked for antenatal care, were referred late or managed beforehand by either traditional birth attendants (TBAs) or faith-based healers. Other deaths actually occurred in transit to hospital or in mission (faith-based) clinics, traditional and domestic homes (Table 2).

**Table 2:** Distribution of Maternal Deaths by Place of Death

Place of Death	2012/2013 Frequency n(%)	2013/2014 Frequency n(%)	2014/2015 Frequency n(%)	Total [2012 – 2015] n(%)
Mission (Faith-based) home	2 (1.7)	1 (1.1)	3 (3.7)	6 (2.1)
Traditional attendant’s home	4 (3.5)	0 (0)	1 (1.2)	5 (1.8)
In transit	1 (0.9)	4 (3.4)	3 (1.2)	8 (2.8)
Domestic home	1 (0.9)	3 (3.4)	1 (1.2)	5 (1.8)
Patent medicine store	1 (0.9)	0 (0)	0 (0)	1 (0.3)
Private health facilities	3 (2.6)	1 (1.1)	5 (6.2)	9 (3.2)
Public health Facilities	101 (88.6)	80 (89.9)	66 (81.5)	247 (86.9)
Unknown	1 (0.9)	0 (0)	2 (2.5)	3 (1.1)
TOTAL	114 (100.0)	89 (100.0)	81 (100.0)	284 (100.0)

Fisher’s exact test - 16.129, *P* Value - 0.221

*P* value < 0.05 for significant difference

In terms of geographical distributions, Akure South and Ondo West (of Ondo Central District), Owo and Akoko Northeast (Ondo North) as well as Okitipupa (Ondo South) being the most cosmopolitan LGAs in their respective senatorial districts had leading numbers of reported cases of maternal deaths. Table 3 illustrates the distributions of maternal deaths by LGA in the State during the period of study.

**Table 3:** Distribution of Maternal Deaths by Local Government Area

LGA	2012/2013 Frequency n(%)	2013/2014 Frequency n(%)	2014/2015 Frequency n(%)	Total [2012–2015] n(%)
Akoko North East	10 (8.8)	15 (16.8)	11 (13.6)	36 (12.7)
Akoko North West	0 (0)	0 (0)	0 (0)	0 (0)
Akoko South East	0 (0)	1 (1.1)	2 (2.5)	3 (1.1)
Akoko South West	2 (1.8)	3 (3.4)	4 (4.9)	9 (3.2)
Akure North	2 (1.8)	0 (0)	1 (1.2)	3 (1.1)
Akure South	31 (27)	21 (23.6)	20 (24.7)	72 (25.3)
Ese-Odo	2 (1.8)	0 (0)	0 (0)	2 (0.7)
Idanre	0 (0)	0 (0)	4 (4.9)	4 (1.4)
Ilaje	2 (1.8)	1 (1.1)	0 (0)	3 (1.1)
Ile-Oluji/ Oke-Igbo	1 (0.9)	2 (2.2)	2 (2.5)	5 (1.8)
Ifedore	1 (0.9)	1 (1.1)	0 (0)	2 (0.7)
Irele	1 (0.9)	0 (0)	0 (0)	1 (0.3)
Odigbo	2 (1.8)	1 (1.1)	3 (3.7)	6 (2.1)
Okitipupa	11 (9.6)	10 (11.2)	3 (3.7)	24 (8.4)
Ondo East	0 (0)	0 (0)	0 (0)	0 (0)
Ondo West	24 (21.1)	28 (31.5)	16 (19.7)	68 (23.9)
Ose	0 (0)	0 (0)	2 (2.5)	2 (0.7)
Owo	25 (21.9)	6 (6.7)	13 (16.0)	44 (15.5)
Total	114 (100.0)	89 (100.0)	81 (100.0)	284 (100.0)

The age range of women most affected in the first and second periods of study was 31 to 36 years compared to 25 to 30 years in the third one. The distribution of maternal deaths by age is shown in Table 4. The parities most recorded were consistently those patients who had between one and four previous births. The distribution of deaths by parity is illustrated in Table 5. The differences in values for the parities were statistically significant while those of places of death and ages showed none.

**Table 4:** Distribution of Maternal Deaths by Age

Age (years)	2012/2013 Frequency n(%)	2013/2014 Frequency n(%)	2014/2015 Frequency n(%)	Total [2012-2015] n(%)
< 19	0 (0)	5 (5.6)	2 (2.5)	7 (2.5)
19 –24	16 (14.0)	14 (15.7)	8 (9.9)	38 (13.4)
25 –30	36 (31.6)	21 (23.6)	32 (39.5)	89 (31.3)
31 – 36	43 (37.8)	35 (39.4)	26 (32.1)	104 (36.6)
=/>37	16 (14.0)	13 (14.6)	8 (9.9)	37 (13.0)
Unknown	3 (2.6)	1 (1.1)	5 (6.1)	9 (3.2)
TOTAL	114 (100.0)	89 (100.0)	81 (100.0)	284 (100.0)

Fisher’s exact test - 15.559, *P* Value - 0.093

*P* value < 0.05 for significant difference

**Table 5:** Distribution of Maternal Deaths by Parity

Previous Deliveries	2012/2013 Frequency n(%)	2013/2014 Frequency n(%)	2014/2015 Frequency n(%)	Total [2012-2015] n(%)
0	40 (35.1)	16 (18.0)	13 (16.1)	69 (24.3)
1 – 4	54 (47.4)	60 (67.4)	58 (71.6)	172 (60.6)
=/>5	20 (17.5)	10 (11.2)	4 (4.9)	34 (11.9)
Unknown	0 (0)	3 (3.4)	6 (7.4)	9 (3.2)
Total	114 (100.0)	89 (100.0)	81 (100.0)	284 (100.0)

Fisher’s exact test - 29.284, *P* Value – 0.000

*P* value < 0.05 for significant difference

## Discussion

To the best of the researchers’ knowledge this was Nigeria’s first implementation of a state-wide maternal death surveillance and response system in the form of a confidential enquiry into maternal deaths. The 33% reduction in MMR in Ondo State from 253 per 100,000 births (1st June 2012 to 31st May 2013) to 170 (1st June 2014 to 31st May 2015) is striking. This finding was in line with the 70% reduction found in a concurrent study that examined a five-year facility-based MMR trend in the busiest maternity centre in Ondo State [11]. These achievements could be attributed to the multi-pronged “Abiye” (safe motherhood) programme instituted by the state government between 2009 and 2015. The programme included free maternal and child health services in all public hospitals in the state, establishing dedicated tertiary facilities for the care of pregnant women and children as well as collaborating with unskilled traditional and faith-based birth attendants to refer uncomplicated labour cases in their care to designated hospitals in exchange for cash incentives.

In comparison, the South African model came into operation in 1998 but, unlike CEMDOS, was strictly facility–based. There was an initial increase in MMR in South Africa from 1998, peaking at 176 per 100,000 live births in 2010. This was followed by a reduction of 13% to 159 in 2011 and further reduction to 147 in 2012 [12]. Morocco, alongside far reaching health interventions, instituted its maternal death surveillance system in 2009 and showed an average annual decrease in MMR of 5% till 2012 [13].

The reduction in number of maternal deaths and MMRs in Ondo State since the introduction of the confidential enquiries was therefore anticipatory as year after year, measures to counter identified causative factors were instituted. To improve quality of care at the facility level, for instance, the identification of post-partum haemorrhage and eclampsia as the major direct causes of maternal deaths led to resources being invested in conducting targeted trainings and clinical drills among relevant staff in all secondary and tertiary hospitals in the state. In addition, meetings were held at community levels between ministry of health officials and commercial drivers’ unions to make vehicles available at odd hours of the day at subsidised rates to minimise delays in labour cases moving from their homes to maternity centres. Furthermore, trained community health officers were provided tricycle ambulances or motor cycles to assist in responding to home distress calls as well as evacuating patients to health facilities.

The major causes of maternal deaths in this study were the same as identified in other similar reports. For instance in Morocco, haemorrhage contributed 33% of direct causes of death followed by pre-eclampsia/eclampsia, infections and uterine rupture [13]. The South African data, however, showed that non-pregnancy related infections and acquired immuno-deficiency syndrome (AIDS), ranging from 54 to 73%, accounted for majority of their maternal deaths between 2002 and 2012 [12]. Thrombosis and thrombo-embolic disorders were found to predominate in the United Kingdom [9]. The identification of causes and surrounding circumstances leading to maternal deaths in any environment should lead to prioritisation and formulation of policies targeted at those particular challenges.

This study showed that about 90% of women died in hospitals though a majority of them had been delayed or managed elsewhere. Unique about CEMDOS is that deaths outside facilities are included. Hitherto, maternal death audits from Nigeria emanated from secondary and tertiary-level hospitals, a majority of which are located in urban or semi-urban areas. All these report contained data confined to individual hospitals, none did collate or compare data across hospitals or within a specified area [14]. Limiting data to facility deaths leads to significant underreporting of deaths in a country like Nigeria. Surveys showed only 35% of Nigerian women gave birth in hospitals [3]. The Moroccan report also took into cognizance events outside hospitals, giving a rate of about 70% facility deaths and 4%, in transit to hospitals [13]. In South Africa, however, their confidential enquiry contained facility-based data only, but in a setting where over 90% of the population gave birth in facilities [12].

With regard to geographical distribution of maternal deaths in Ondo State of Nigeria, the sharp drop of about 35% in Akure South compared to other LGAs in the initial 2 years was notable. This particular feat may be attributed to implementation of the home-grown “Agbebiye” initiative, a component of the “Abiye” programme, which involved registration of all TBAs in this LGA and establishing an incentive-based system for them to refer women in labour to hospitals before complications set in. In exchange each TBA received a payment voucher per referral to be redeemed at a later date.

In addition, entrepreneurship workshops were organised and seed money distributed to allow registered TBAs seek other vocations like catering, bead-making and soap production as alternative means of livelihood. The impact on number of reported deaths was so impressive that the initiative was replicated in phases, first in Ondo west then the other LGAs. Geographical pattern of maternal deaths also resulted in targeted health interventions in South Africa especially in Free State province that initially had relatively high numbers of reported deaths [12]. A three-pronged strategy involving inter-facility ambulance transport, intensive district training, and re-alignment of hospitals performing surgery contributed to a reduction [15].

The Ondo State pattern revealed that LGAs like Akoko Northwest, Ese-odo and Ilaje, which habour a predominantly rural population and difficult terrains, recorded paradoxically low numbers of reported deaths. This might have been due to under-reporting as a result of poor communication infrastructure as well as gravitation of complicated labour cases towards more developed LGAs in the State. There was evidence of ineffective integration of the CEMDOS scheme into existing but deficient structures in these areas like the radio, television and mobile networks made worse by the pervasive lack of electricity. These anticipated teething problems were limitations to the study as with any new health initiative requiring public participation. Additional interventions like reconnecting the areas to the national electricity grid must be instituted to address these specific challenges, in order to generate more accurate data.

This study showed that the overall age range-at-risk was 25 to 36 years accounting for an average 68% with no discernible trend. The Moroccan study also showed nearly 50% of the women were between 25 and 35 years old. In addition, they had on average, two children at time of death [13]. Similarly in this study, the parities most recorded were consistently of women with 1 to 4 previous births. This may point to an overall disproportionately large number of parturients falling in that parity range rather than suggesting they were at higher risk of maternal mortality. It is pertinent to note that a majority of maternal deaths were preventable and occurred when most women were contributing maximally to the socio-economic development of their households in particular and nation, as a whole. The implementation of a maternal death surveillance and response system as exemplified in this study by a state-wide confidential enquiry format appears to be a pre-requisite to combating this tragedy of our time.

Our recommendations for replication of Ondo State model include; utilisation of available employees (i.e. disease surveillance and notification as well as monitoring and evaluation officers) at the local government level as well as in hospitals at no significant extra cost. The limited resources could then be majorly expended on logistics and creating public awareness like the use of jingles in electronic media as was done in Ondo. In addition, there should be a focus on addressing delays in seeking and reaching care among parturients through the provision of vehicular means of transport to and from health facilities. Furthermore, collaborating with stakeholders at community level, particularly the TBAs have been shown to be worthwhile. Finally, improving quality of care in facilities through capacity building for relevant healthcare professionals in order to combat the leading causes of maternal deaths would go a long way to reduce deaths from complications of pregnancy, labour and puerperium.

## Conclusion

There was a trend of a reduction in maternal mortality ratio during the period of study with postpartum haemorrhage as the major cause of death. This reduction, combined with the positive outcomes of interventions at local government levels as well as other factors enables us to recommend that our confidential enquiry model be adopted in other states and at the federal level. It should be a matter of best practice to routinely perform careful analysis of maternal deaths in a systematic and robust manner.

## Data Availability

The datasets generated and analysed during this study are not publicly available due to their sensitivity and the need to ensure confidentiality but these are available from the directorate of planning, research and statistics of the Ondo State ministry of health upon reasonable request.

## Abbreviations

AIDS: Acquired immuno-deficiency syndrome
CEMDOS: Confidential Enquiry into Maternal Deaths in Ondo State
CI: Confidence interval
LGA: Local government area
MMR: Maternal mortality ratio
PPH: Postpartum haemorrhage
SPSS: Statistical Package for the Social Sciences
TBA: Traditional birth attendant
WHO: World Health Organisation
